# Molecular musings in microbial ecology and evolution

**DOI:** 10.1186/1745-6150-6-58

**Published:** 2011-11-10

**Authors:** Rebecca J Case, Yan Boucher

**Affiliations:** 1 Department of Biological Sciences, University of Alberta, Edmonton, AB, T6G 2E9, Canada

**Keywords:** Ribosomal RNA genes, diversity, lateral gene transfer, microbial ecology, microbial evolution, evolutionary microbiology, ecological microbiology, Tree of Life

## Abstract

A few major discoveries have influenced how ecologists and evolutionists study microbes. Here, in the format of an interview, we answer questions that directly relate to how these discoveries are perceived in these two branches of microbiology, and how they have impacted on both scientific thinking and methodology.

The first question is "*What has been the influence of the 'Universal Tree of Life' based on molecular markers?" *For evolutionists, the tree was a tool to understand the past of known (cultured) organisms, mapping the invention of various physiologies on the evolutionary history of microbes. For ecologists the tree was a guide to discover the current diversity of unknown (uncultured) organisms, without much knowledge of their physiology.

The second question we ask is "*What was the impact of discovering frequent lateral gene transfer among microbes?" *In evolutionary microbiology, frequent lateral gene transfer (LGT) made a simple description of relationships between organisms impossible, and for microbial ecologists, functions could not be easily linked to specific genotypes. Both fields initially resisted LGT, but methods or topics of inquiry were eventually changed in one to incorporate LGT in its theoretical models (evolution) and in the other to achieve its goals despite that phenomenon (ecology).

The third and last question we ask is "*What are the implications of the unexpected extent of diversity?" *The variation in the extent of diversity between organisms invalidated the universality of species definitions based on molecular criteria, a major obstacle to the adaptation of models developed for the study of macroscopic eukaryotes to evolutionary microbiology. This issue has not overtly affected microbial ecology, as it had already abandoned species in favor of the more flexible operational taxonomic units. This field is nonetheless moving away from traditional methods to measure diversity, as they do not provide enough resolution to uncover what lies below the species level.

The answers of the evolutionary microbiologist and microbial ecologist to these three questions illustrate differences in their theoretical frameworks. These differences mean that both fields can react quite distinctly to the same discovery, incorporating it with more or less difficulty in their scientific practice.

**Reviewers:**

This article was reviewed by W. Ford Doolittle, Eugene V. Koonin and Maureen A. O'Malley.

## Background

We often think of how certain major scientific discoveries have affected our own scientific field. What we more rarely think about is that such discoveries might have impacted other fields in completely different ways. Here, we have identified three discoveries we think have had critical impacts on microbiology during the past two decades. Interestingly, two branches of microbiology have been impacted very differently by these discoveries. Although the fields of evolutionary microbiology (the study of microbial evolution) and ecological microbiology (known by its practitioners as microbial ecology) somewhat overlap each other in their subject of inquiry, they have very distinct views on several biological processes occurring in microbes.

Evolutionary microbiology can be defined as the study of the patterns (relationships between genes and organisms) and processes (mechanisms generating diversity and the selection operating on it) of evolution in microbes. Its main tools are reconstruction of relationships (using phylogenies and networks), population genetics, and comparative gene and genome analyses. Microbial ecology is the study of microbes' relationship with their environment and other organisms. Microbial ecology has historically focused on the relationship microbes have in host systems as they form beneficial liaisons (e.g. nitrogen fixing rhizobia in plant root nodules) and pathogenic relationships (e.g. *Pseudomonas aeruginosa* in the lungs of people with cystic fibrosis) with their hosts. The other focus has been the ways in which microbes drive the nutrient cycles that make earth habitable for humans. More recently, microbial ecology has borrowed theoretical ecology from macroecology and adapted it to microbial systems (e.g. landscape ecology, keystone species, biogeography) [1-3].

The first of three major discoveries we will discuss as having had an impact on these two branches of microbiology is that molecular markers can be used to reconstruct a phylogeny of all organisms (a 'Universal Tree of Life'). This tree, regardless of its theoretical validity, has served as a framework to scientific inquiry in microbiology for decades. The second discovery is that lateral gene transfer (LGT), the acquisition of genetic material by means other than inheritance from a progenitor, is frequent among bacteria and archaea. By disconnecting the history of genes from that of the organisms carrying them, LGT fundamentally affected how we can describe bacterial and archaeal evolution and ecology [4]. The third discovery is that the extent of genetic diversity in microbes is much greater than expected and can vary between species [5,6], which brings into question the universality of species as a unit of diversity or evolution.

These discoveries have all been examined to some extent previously. Here however, we look at how two different fields, those of evolutionary microbiology and microbial ecology, interpret these discoveries, and assess how they have affected their methodology and subjects of inquiry. To do so, we ask a question pertaining to each discovery in an interview format and present the answer from both evolutionary and ecological perspectives.

## Discussion

### What has been the influence of the 'Universal Tree of Life' based on molecular markers?"

Carl Woese's work established ribosomal RNA (rRNA) as the reference molecular marker by cataloguing and comparing rRNA molecules in order to determine evolutionary relationships between organisms, even before the widespread use of DNA sequencing [7]. This led to the discovery of a completely new domain of life, the Archaea [8]. When it became possible to sequence DNA, small ribosomal subunit gene (16S rRNA gene) sequences were used by Woese to reconstruct the 'Universal Phylogenetic Tree', now more commonly known as the 'Tree of Life' [9]. The idea that there was a unique tree describing the relationship between all organisms quickly took hold in microbiology. However, it influenced evolutionary and ecological branches of microbiology in different ways.

#### Evolutionist

For evolutionists, knowing when and where major evolutionary inventions occurred has always been of great interest. Even in the 1980s, it had been known for decades that microbes tolerated extreme environmental conditions (temperature, salinity, radiation, etc) and performed a variety of metabolic reactions (photosynthesis, sulfur oxidation, methanogenesis, etc) [10]. However, when they had evolved could only be extrapolated from the geological record for some of these physiological characteristics, and the groups of organisms in which they evolved mostly remained a mystery. The 'Universal Phylogenetic Tree' proposed by Woese would allow evolutionary microbiologists to find where these inventions belonged in the history of life. Woese and others continued to use gene sequences as tools to gather information about when and in which group key evolutionary events or inventions happened. For example, we realized that photosynthetic bacteria were not monophyletic [7] and that methanogenesis is an ancient invention in the Euryarchaeota phylum of the Archaea [9]. The relationships between groups of microbes previously thought of as unrelated could also be established, such as the monophyly of Flavobacteria and Bacteroides [11] or of Deinococcus and Thermus [12]. Very importantly, rRNA gene sequences were key data in identifying the bacterial origins of mitochondria and chloroplast [13,14]. Although this is widely contested, the 16S rRNA gene tree has also been used to infer characteristics of the common ancestor(s) of bacteria, such as thermophily, based on the deep branching of the thermophilic *Thermotoga *and *Aquifex *[15]. Woese's construction of the 'Tree of Life' established the groundwork for a molecular revolution in evolutionary microbiology, giving us the phylogenetic backbone that allowed specific lineages to be linked to given physiologies. The meaning of an rRNA-based 'Tree of Life' has been seriously questioned, as it depicts the evolution of a single gene, not the whole genomes of organisms [16]. Regardless of its meaning, however, such a tree focused the attention of evolutionary microbiologists to the phylogenetic mapping of physiological innovations in Bacteria and Archaea. This was mostly done using molecular data from known organisms found in culture collections, without much consideration for their ecology: where they are found in nature and how they made a living.

#### Ecologist

In microbial ecology, a 'Phylogenetic Tree of Life' based on 16S rRNA genes inspired a taxonomic tool for the identification of microbes in order to infer physiologies from a gene sequence, rather than one for mapping the invention of physiologies. In 1990, two teams of microbial ecologists published the first 16S rRNA gene clone libraries from the Sargasso Sea and hot springs in Yellowstone National Park in the same issue of Nature [17,18]. This method was revolutionary, as clone libraries allowed sequencing of 16S rRNA genes amplified directly from environmental DNA, thereby avoiding the requirement for cultivation of their host. The Sargasso Sea study depicted its sequence data in a phylogenetic tree, including sequences from cultured organisms, allowing researchers to identify previously uncultured bacteria from the environment. Their depiction of sequence data in a phylogenetic tree was reminiscent of Woese's 'Universal Phylogenetic Tree' [9], as was their choice of molecular marker, which was guided by his previous work. As trees allow microbial ecologists to assign 16S rRNA gene sequences to phyla, genera and occasionally species, and reveal previously unknown groups, diversity became a central theme in microbial ecology. Novel groups with no cultured representatives were the most intriguing result from early diversity studies, as these groups outnumbered those which were known from cultured isolates [19]. Even today, most of these novel groups, usually called candidate phyla or divisions, still have no cultured representative. This is the case for many of the thermophilic candidate divisions discovered in Obsidian Pool in Yellowstone National Park [20]. However, due in large part to the efforts of Giovanonni and his team, there are now cultured representatives for some of these elusive 'unculturable' organisms (e.g. SAR11) [21]. For most microbial ecologists however, these initial studies did not lead to further culturing efforts, but to a quest to fill in the missing branches of the 'Tree of Life' [19]. This quest is reflected in the fact that over 2000 references have been made to the software package, ARB, which allowed researchers to add their sequences to a curated 'Tree of Life' [22]. This extensive sequencing effort by microbial ecologists maintained diversity as a key theme in microbial ecology into the 21^st ^century, as environmental gene clone libraries are constructed for every model system that could be sampled, in fields ranging from biological oceanography [23] to art preservation [24]. Consequently, the 16S rRNA gene is the most sequenced of all genes. Previously unknown groups of organisms can now be studied directly in the environment using molecular techniques and often prove to be abundant, confirming what microbiologists had long suspected; Prokaryotes are the unseen majority [25]. However, our physiological and genetic understanding of bacteria and archaea is based on domesticated strains that are unlikely to tell us much about the untamed majority driving earth's biogeochemical systems. This realization in part drives the larger metagenomic, metaproteomic and metatranscriptomic efforts in microbial systems. The preoccupation with measuring the diversity of microbes has also been at the expense of understanding the theoretical forces that shape microbial communities, although some theoretical ecology studies are now being applied to microbial systems.

### What was the impact of discovering frequent lateral gene transfer among microbes?

Although it has been clear that genes could be exchanged between bacteria through LGT as early as the 1950's [26], it took a long time for this phenomenon to be recognized as important in their evolution and ecology. This is likely related to how useful the 'Tree of Life' had been in identifying Bacteria and Archaea, describing their diversity, and linking groups of microbes to specific physiological functions. With the advent of genomics however, it became increasingly clear that a large portion of genes in many bacterial and archaeal lineages had been acquired through LGT, sometimes consisting in 20-30% of their genome [27,28].

#### Evolutionist

The fact that LGT had contributed so much to the genomes of some bacteria and archaea was a turning point for evolutionary microbiologists, who started (slowly) changing their practice, both in what they study (level of biological organization) and how they study it (methodologies). The high frequency of LGT among microbes certainly brought many doubts to the practice of increasing phylogenetic signal by using multiple genes (sometimes hundreds) to construct a tree [29]. If these various genes display conflicting phylogenetic signals, the result is a tree that does not represent the real history of cells, but rather some average of their components various histories. This is progressively leading to the use of alternative methods for depicting the evolution of microbial cells, such as genetic networks, a graph-theoretical representation that can include non-vertical histories [30]. The complexity of constructing and interpreting such networks has led to a persistent (although more careful) use of trees, but also to the adoption of more simple measures of similarity, such as average nucleotide identity, average amino acid identity or gene content similarity, often illustrated as simple graphs [31,32]. Besides an impact on the methodology used to infer the evolutionary relationship of organisms, there has been a movement towards looking at the evolutionary history at different levels of biological complexity besides organisms. Evolutionists are now interested in looking at the history of elements found at lower levels of complexity, such as genes, pathways or genomic islands, learning about their origins, dissemination patterns and rates of change [33]. Evolutionary microbiologists are also directing their attention to higher levels of biological complexity, such as that of populations (through multi-locus sequence analyses (MLSA) or whole genome sequencing of hundreds of strains from a given population), at which recombination or LGT are relatively easily illustrated using networks or statistical analyses [34].

#### Ecologist

For microbial ecologists, it was the realization that they could not accurately predict the ecological function of a microbe from its 16S rRNA gene sequence that led to the recognition of the importance of LGT [35]. However, very few microbial ecologists changed their practice, and most increased their focus on questions in which the effects of LGT was not clear (i.e. diversity estimates based on a single marker gene) or set limits on the interpretations of results they obtained. The importance of this phenomenon was also initially resisted [36,37] and its impact was often marginalized as only pertaining to a unique minority of organisms, possibly in part because of the extensive use of 16S rRNA gene-based methodologies (clone libraries, denaturing gradient gel electrophoresis (DGGE), restriction fragment length polymorphism (T-RFLP), fluorescent in-situ hybridization (FISH)). These methodologies require a stable association of 16S rRNA genes with specific metabolic traits or behavior to make the crucial link between the composition and function of microbial communities. It became clear that with the presence of LGT, functional genes were not necessarily associated with specific 16S rRNA gene sequences. This link between the composition of a community (form) and its function was a promise derived from macroecology. In that discipline, based on our understanding of the vertical inheritance of traits, we can confidently predict that a Coniferales is photosynthetic; however the same cannot be said of the Roseobacterclade, where some species of the genus are photosynthetic and others are not [38]. This idea was central to the initial interest of molecular microbial ecology to many other fields (e.g. earth sciences, oceanography, agricultural sciences and medicine). It also offered a 16S rRNA gene-based molecular toolbox that would allow the identification of the microbes responsible for the processes studied, from carbon remineralization to polymicrobial diseases. In this vision, once an organism's 16S rRNA gene sequence was obtained, its traits could be inferred from a tree generated using the software ARB (from arbor) [22] and it could be tracked in the environment using the polymerase chain reaction or FISH [39]. As the association between specific functions and branches of the 'Tree of Life' was continually put into doubt by LGT, many scientists were inadvertently forced to question whether 16S rRNA gene-based molecular tools could answer their questions. The sentence should read: "Researchers started applying methodologies developed using the 16S rRNA gene, such as clone libraries [40] DGGE banding patterns [41], or FISH on the genes encoding the function they wanted to study. This approach has had mixed success due to the inherent bias of primer design for protein-encoding genes [42]. Yet, the scientific focus of microbial ecology remains on community function and diversity, so all-encompassing methodologies that embraced the metagenome, the genetic potential of a community, were rapidly adopted. Although it could be viewed as looking for a needle in a haystack, metagenomics promised to both identify organisms within a community and their functional potential. However, only low diversity systems in which genomes can be reconstructed [43] and large insert libraries [44] have actually managed to link form to function. Metagenomics has since moved into metatranscriptomics and metaproteomics, methodologies that identify the expressed or functioning part of the metagenome [45,46]. The fact that these new methods are not susceptible to LGT (as they analyze data at the level of the gene, not the organisms) might have played a role in how rapidly they were adopted at the expense of traditional 16S rRNA gene based methodologies.

### What are the implications of the unexpected extent of diversity?

Through the combination of comparative genomic studies [3], MLSA (multi-locus sequence analysis) of bacterial populations [47] and 16S rRNA gene tag sequencing with high-throughput technologies [48], it has now become evident that microbial populations are much more diverse than previously thought. Furthermore, the extent of diversity varies significantly between species defined using traditional criteria [49]. This has important implications for both evolutionary and ecological microbiology.

#### Evolutionist

Two key pieces of research clearly demonstrated that genetic diversity could be far larger than we had ever imagined in bacteria and archaea. The first evidence for such diversity came out of one of the earliest comparative genomics study, in which the gene content of three *Escherichia coli *genomes was compared, showing that any two strains shared only about 40% of their genes [50]. This was later confirmed on a much larger scale in the marine heterotroph *Vibrio splendidus *[51]. It was shown that a few dozen milliliters of seawater could contain hundreds of genotypes of that species that differed by up to 20% in genome size. Although the biological implications of this diversity are still unclear, its extent is strongly suggestive that the species, as defined today, is not a finely resolved unit of diversity, because it can encompass a range of organisms that are genetically distinct [3].

This unexpectedly high diversity at the species level or below forced evolutionary microbiologists to abandon the use of low-resolution molecular markers such as the 16S rRNA gene in favor of others methodologies to describe relationships between isolates. The most popular of these has been MLSA. The amplification and sequencing of multiple housekeeping protein-coding genes (usually six to eight) to identify bacterial isolates is now widespread in microbiology [52]. The larger number of markers used, as well as the higher sequence variability of protein-coding genes, provided evolutionary microbiologists with a high-resolution method to identify relationships between isolates below the species level. This technique also allowed us to measure which part of the observed genetic diversity in a population of isolates was caused by point mutation and which part was caused by recombination. A wide variation in the importance of these two sources of genetic diversity was observed between different species, and even within species [53]. This was the first evidence that the unexpected extent of genetic diversity could be partially explained by the fact that it originated from multiple processes. It rapidly became obvious that not only the processes giving rise to nucleotide substitutions (mutation and recombination) contributed to the extensive genetic diversity observed in microbes, but also those altering their gene content. For example, the main source of genetic diversity in the causative agents of scrub typhus, *Orienta tsutsugamuchi*, are integrative conjugative elements and transposable elements [54]. The most variable region of *Vibrio cholerae *genomes are integrons [55], and the aphid symbiont *Buchnera aphidicola *mostly evolves through nucleotide substitutions as well as small insertions and deletions [56]. Therefore, not only is genetic diversity much more extensive than expected, but its extent varies from one species to another. For evolutionary microbiologists, a variation in the extent of diversity across organisms made a universal definition of species based on molecular criteria impossible. The lack of such a definition makes the development of population genetics for bacteria and archaea very challenging, as most of the theory behind this branch of biology is based on having a species definition [47]. Without a species definition, there cannot be a single mathematical model to determine what composes a separate population or what form of selection is operating on a population. This also has far reaching consequences for taxonomy, because even though organisms can be classified in species using any *ad hoc *molecular criteria (i.e. 95% average nucleotide identity for protein encoding genes or 98% 16S rRNA gene sequence identity), it does not mean these species will have any biological meaning.

#### Ecologist

To officially describe a novel bacterial or archaeal species, a representative must be available in pure culture, so that biochemical tests and DNA-DNA hybridization can be performed to compare it to known species (although this can now be done indirectly by sequencing its genome). This has long been problematic for microbial ecology, as most microbes are not in culture and many of our methodologies require a measurable unit of diversity to be calculated. For this reason, units other than species have frequently been used. It should be noted that there has been one remarkable study which used the traditional species definition to determine the number of bacterial genomes in a soil sample by making a melting curve of DNA extracted directly from the environment [57], demonstrating the immensity of bacterial diversity. However, this methodology was too technically challenging to be broadly adopted. To circumvent problems associated with using species as units of diversity, the concept of Operational Taxonomic Unit (OTU) is employed. An OTU is an arbitrary unit commonly used in microbial ecology as its definition can be specific to a method, such as a unique chromatogram peak for T-RFLP, a band within a DGGE pattern, or what was most commonly used for isolates or in clone libraries, 97% or greater sequence identity between 16S rRNA gene sequences. These practical definitions allowed ecological parameters such as evenness and species richness to be calculated and microbial communities from different samples to be compared. The drawback is that the OTUs are so method-dependent that comparisons between studies are problematic. Also, a growing number of examples of speciation within the 97% cut off were being identified [31], revising up the value to ≥ 98% sequence identity [58]. Acinas and Klepac-Ceraj *et al*. [59] later demonstrated that every nucleotide substitution in the 16S rRNA gene could represent significant diversity. The extent of diversity below the species level was therefore enormous and the dropping cost of sequencing allowed researchers to adapt by simply sequencing more, such that lineages representing individual sequences began to be commonly replaced by wedges in the tree [19] and communities (groups of sequences) were now condensed into a matrix and compared as groups [60]. The biggest increase in the number of 16S rRNA gene sequences came with the advent of next-generation sequencing, as tens of thousands instead of hundreds of sequences could now be generated at a comparable cost. The irony is that these sequences are shorter and of poorer quality than those that can be obtained through traditional Sanger sequencing. This makes sequence identification possible only at the genus level or above and what may be newly discovered diversity is often discarded as we are unable to confidently place sequences in the 'Tree of Life' [61].

The extensive diversity at or below the species level, as we have just seen, is not yet well captured by the main methods used by microbial ecologists. The proportion of such diversity that is linked with ecological function is not known, but there is strong evidence for phenotypic differentiation occurring below the species level. Such differences can be observed in *E. coli*, whose various subspecies cause different diseases or occupying different niches [62]. The genus *Shigella*, for example, are in fact a polyphyletic group entirely found within the *E. coli *species. It is defined based on the fact that it can cause shigellosis, and whose pathology is due to a set of virulence factors found on a plasmid [63]. This diversity within a species is also found in environmental microbes, for example the cyanobacterium *Prochlorococcus*, which is a genetically coherent group which roughly corresponds to a traditional species, in which all members share > 97% 16S rRNA gene sequence identity. It is one of the most abundant photosynthetic organisms in the ocean, containing various ecotypes specialized to live at different light intensities, which correspond to different depths in the ocean [64].

Although microbial ecology has yet to find a way to deal with the extensive diversity below the species level, it has not been concerned by the fact that the extent of diversity varies widely between taxonomic groups. This is mostly because microbial ecologists have long abandoned the dream of a universal molecular species definition in favor of a more practical one, the OTU. Since the definition of an OTU can vary between datasets, it can be adjusted to match the level of diversity or marker employed. However, if most diversity is generated through LGT, a single molecular marker such as 16S rRNA gene, no matter how stringent the OTU definition, will not necessarily capture it. The hope of microbial ecologists is that this diversity can be captured using metagenomics and the functions associated with this diversity through metatranscriptomics and metaproteomics.

## Conclusion

Both evolutionary and ecological microbiology were using the 'Tree of Life' as a tool to guide their scientific inquiry, creating a common focus within these branches of microbiology. However, the tree was placed inside different theoretical frameworks for these two fields. The tree was a tool for the evolutionists to understand the past (history of evolutionary inventions), while it was one to understand the present for ecologists (current diversity). Evolutionists focused on understanding how the physiology of known groups of cultured organisms came to be, while ecologists looked at where unknown organisms existed with little understanding of their physiology.

Both evolutionary microbiology and microbial ecology initially resisted the discovery that LGT is frequent in microbes, as it made their respective 'holy grails' inaccessible. For evolutionary microbiology, there would never be a simple and unique depiction of relationships between organisms, and for microbial ecologists, functions could not be easily linked to specific genotypes. After initial resistance to the importance of the phenomenon [65], evolutionary microbiology actively changed its practices, developing methodologies and models that made lateral links between organisms possible and focusing investigation at levels of biological organization below or above the organism (gene or population), which are methodologically more amenable to the existence of LGT. Ecological microbiology developed novel methodologies that allow links to be established between community structure and function (such a metagenomics), even if LGT is rampant between organisms, as they examine molecular data at the gene or population level.

The unexpected extent of diversity forced evolutionary microbiology to adopt new approaches providing higher resolution to define relationships between organisms. The use of these techniques, such as MLSA, uncovered the fact that the level of genetic diversity varies between species. This directly affected evolutionary microbiology, invalidating the universality of species definitions based on molecular criteria. Microbial ecology, on the other hand, relied heavily on technology to deal with the extent of diversity, hoping that more data would resolve this issue. However, the main molecular marker used (16S rRNA gene) lacks resolving power at or below the species level, missing where most of the unexpected diversity lies. Furthermore, the high error rate of sequences obtained with new technologies prevents accurate estimates of diversity [66]. The hope is that combining these new technologies with new approaches that do not rely on a specific marker (such as metagenomics) will allow more accurate estimates of diversity. The discovery that levels of genetic diversity vary across taxa did not affect microbial ecology as it did evolutionary microbiology, as this field has widely given up the usage of species as biological units in favor of OTUs, which can be defined based on the dataset being analyzed and are by definition not universal.

Overall, we can note that although they share microbes as their subject of inquiry, the theoretical frameworks of evolutionary microbiology and microbial ecology are quite different (one being inherited from evolutionary biology and the other from macroecology). Consequently, the same discovery is not always integrated as easily into both fields. When a discovery is not easily accommodated in the existing framework, there seems to have been three strategies: change the methods, change the questions, or 'wait and see'. It seems that with enough work, most important discoveries can been integrated through a change in methods or questions, but that a 'wait and see' period is sometimes necessary before arriving there.

## Competing interests

The authors declare that they have no competing interests.

## Authors' contributions

Both authors contributed equally to the planning and writing of this Review. The authors have also both read and approved the manuscript.

## Reviewers Comments

**Reviewer 1: **W. Ford Doolittle (Dalhousie University, Halifax, Canada)

This is a nice breezy review of the current state of microbial ecology and evolution. I very much like the Q and A approach, since it highlights disciplinary differences. It would be nice to see someone (not necessarily these authors, and of course not right now) rewrite the history of the last 50 years of molecular phylogeny (its whole history) in terms of the biases imposed on its leading practitioners by their scientific training. Would we have 16S rRNA as the hegemonic "molecular chronometer" had Woese not spent much of his early career studying the code, translation and the ribosome? Had that not happened, and had our thoughts about species and their relationships been guided more by developments in genetics and epidemiology than in phylogenetics and origins-of-life chemistry, would we be so keen to see meaning in any universal "Tree of Life"? Had microbiologists paid more attention to philosophers, and indeed to Darwin, would we still be asking ourselves what microbial species are? I like to imagine that we could somehow disentangle all the "facts" we know about microbes from the theories (including biological evolution) by which we understand such facts and the histories (of our science) that we use in presenting and arguing these theories. We would then lay just the facts out before someone entirely ignorant of and maybe even hostile to all the rest - a Martian perhaps, or some Republican presidential candidate - and ask them to come up with some useful generalities. It's fun (and good science) to try to figure out what these generalities might be.

**Author's response: **We thank the reviewer for this interesting insight on how our fields would be different if some of the key players had different training or interests or if we had adopted different perspectives. This could be very illuminating, as it would likely highlight certain factors that play a role in directing the questions we ask and the ideas that are retained by the scientific community.

**Reviewer 2: **Eugene V. Koonin (NCBI, NLM, NIH, United States)

In this essay, written in the unusual format of an interview with fictional, generic 'Evolutionist' and 'Ecologist', Case and Boucher discuss the concepts of Tree of Life and species in the light of extensive HGT that is now universally recognized as a major modality of evolution in the prokaryote world. The article is indeed written in the 'musing' style so that no particularly strong or unusual statements are offered. That being said, it took me by surprise that Case and Boucher consider the main impact of the discovery of massive HGT to be on the species concept that they contend to be rendered irrelevant for prokaryotes. I tend to disagree with this view on more than one account. First, I do not think that the species concept is of such overarching importance. Surely, it has been used over many decades to describe relationships between sexually reproducing organisms in a convenient, straightforward way. However, as Darwin was the first to presciently note in the Origin, species is only a convention not really an object, and in that sense, although the species concept has been operationally enormously useful throughout the history of biology, it hardly is 'fundamental'. Second, be as it may, it is rather disingenuous to claim that the species concept is irrelevant for prokaryotes. The most useful definition, pioneered by Mayr, describes species as a group of populations that intermix with each other but are genetically isolated from other populations. From that perspective, prokaryotes are quite diverse: studies on population structure and dynamics show that some are nearly clonal and hence form reasonably good species whereas others constantly exchange genes with diverse partners and so do not fit the species definition (this complexity is well captured in the article of Doolittle and Zhaxybayeva).

**Author's response: **We agree with two of the reviewers that the issue concerning species definitions (the operational criteria for defining a species, such as 98% sequence identity in the 16S rRNA gene) or concepts (theory-based description of species) is not that they are not valid, but rather that they are not universal. We have clarified the text concerning this issue. We now explain that this lack of universality is in part due to the fact that even when using a simple species definition based on a identity cutoff for a marker gene or an average of the genes in the genome, the diversity found in each species can vary extensively, as can their ecological characteristics, meaning that they do not necessarily have any biological meaning. It is the species category, not definitions or concepts, which is not applicable to prokaryotes, as the latter corresponds to a universal species concept is based on biological characteristics.

**Reviewer 3**: Maureen A. O'Malley (University of Sydney)

It is a useful and potentially illuminating exercise to compare how evolutionary and ecological microbiology have taken up molecular phylogenetic approaches under the banner of the Tree of Life. The authors are to be commended for their efforts to think about these issues in an innovative format. However, for this essay, more work has to be done to bring out a clearer and more sophisticated picture of the differences in these fields, and what the implications are for future research.

I will suggest below some elaboration and rethinking for the main body of the text, all of which will require less vague wording in the opening 'Discussion' outline. In particular (in the Discussion outline), it is not clear what is meant by a 'reductionist' approach to defining species, and there is an implication that a focus on the species or genera level is itself reductionist. Wherever 'microbial evolution' is used throughout the text, the authors mostly mean 'evolutionary microbiology' (the field, not the phenomenon) and should revise accordingly. I suggest the same for 'microbial ecology': ecological microbiology. The 'Conclusion' both in the opening outline and the final conclusion to the main text should be elaborated. They need stronger analysis and summing up to make the paper conclude with bolder statements. Overall, this is what needs to be worked out in the paper: the differences between evolutionary and ecological microbiology should be clearly established if there are any. They should be assessed for their importance and used to draw some kind of 'moral' for these fields and perhaps microbiology (or even biology) as a whole. Otherwise, the paper seems to repeat fairly well known facts without taking them further - certainly not 'beyond the tree of life'.

*Background section*. It does not seem to me that three 'concepts' have been identified. The first, 'Woese's work to construct a TOL' is discussed as a method. How has it 'had more impact on the scientific method than any other discovery in microbial ecology and evolution'? What is meant to 'the scientific method' here anyway? Why is LGT a concept when it is described as a natural phenomenon? And likewise, 'intraspecies diversity'? I suggest that more work is done here to sort out what the relevant concepts are, if they are concepts, followed by a straightforward outline of exactly their importance for microbiology without presupposing your subsequent analysis. Simply state why your three factors have implications both for understandings of evolution and ecology.

*Question One: What do you see as the impact of Woese's work on the Tree of Life? *I think this question is not phrased correctly. It should probably be discussed as what some of the perceived shortcomings are of trying to reconstruct the universal tree of life on the basis of molecular markers when these are conceived of as telling a unitary story. While Woese can be 'blamed' or praised for getting this effort going, and for trying to re-conceptualize the field out of its problems, it is almost like saying 'imagine a revolution' without the revolution if the question is asked in this way. Again, it is not at all clear to me why efforts to use molecular markers are 'concepts' and not methods, so clarification is needed at the end of the evolutionist's response to this question. For the ecologist's answer, wasn't diversity always the central theme in ecological microbiology? Is there evidence to support the claim that only with molecules did diversity become an issue for ecological microbiology? I found the concluding sentences of the ecologist's response very hard to follow. It would be good to spell out what is being said here, and to try to come up with a statement about ecological microbiology that connects logically with the discussion about molecular techniques, shows what the problems are, and then how the issues are different from the evolutionist's (if they are). I don't understand what is being implied by 'rethinking microbiology textbooks' so this needs clarification.

*Question 2: Did the discovery of LGT influence thinking in your fields? *I fear this is a rather obviously answered question (and almost banal in that everyone knows what the answer will be). What you need to sort out here is a more multidimensional query about problem: *how *has LGT been a problem for evolutionary microbiology, and is it a different problem for ecological microbiology? For the sake of this style of paper, you probably need to emphasize the differences, or the innovative split format (evolutionist versus ecologist) is not going to be worth it. The evolutionist argues that 'the recognition of LGT as a major evolutionary force' (this is the concept, rather than the phenomenon - see above) had its most fundamental impact on the species concept. This is probably a mistake. There are massive problems with species concepts that are independent of LGT, and placing the problem simply as the species issue does not bring out the conceptual and methodological issues of LGT in anywhere near enough a forceful manner. It is also worth being careful about just where in the biological world LGT is a major evolutionary force. The key problem in this section is going to be saying anything that hasn't been said thousands of times before, so it is imperative to try and do something a bit less run-of-the-mill in the evolutionist's section.

For the ecologist's response, it would be good to have some examples of ecological microbiologists 'resisting' LGT. The examples from plants and animals about vertical inheritance of traits are not helpful for allowing the reader to understand the issues in microbial ecology. Remember, the very issues that made it difficult for microbiology to be regarded as a science in the 19th century revolved around whether microorganisms had specificity (specific effects that were predictably heritable). How and when does it come about in ecological microbiology that it has 'allure' for 'many other fields' because of cast-iron relationships between microbial form and function? Clarification is needed here. The shift into metagenomics also needs to be done more carefully, especially to demonstrate the communities are the level on which all ecological microbiology focuses (not population, for example). How are metatranscriptomics and metaproteomics 'revealing a Tree'? And why is it 'more intricate and beautiful, beyond anything we imagined'? Because it is not a Tree? It would be worth adding references and discussing some of the issues that arise when metatranscriptome and metaproteome data are used for tree-building purposes. I think the concluding sentence of this response has to be rethought entirely, and a tighter, more contrastive ending found for this section.

*Question 3: Has the extent of intraspecies diversity made species an inadequate unit of diversity? *This question should also be rephrased. Putting it in Y/N form makes it seem oversimplified and question-begging (presupposing the conclusion). Perhaps setting up a question that asks 'how' would help, but I worry that it might be the wrong question altogether. At least justify somewhere why intraspecies diversity is your final theme, rather than the dozens of other phenomena that could have been discussed. When the evolutionist answers, he says that MLSA shows the inadequacy of the species concept, but it seems that the method is the problem. The different causal processes are really important, and quite probably of major importance to evolutionary and ecological understanding, so it may be worth putting them as the object of the question rather than 'intraspecies diversity'. The issue is not that it is impossible to 'define species based on molecular criteria': it clearly is and has been possible. The issue is universality. Some more reflection on the evolutionary processes, patterns and theoretical consequences would be useful for this question.

In the ecologist's response, there is a reiteration of 'more diversity than expected', but not much is done with this. Is the problem really that there is so much diversity it cannot be 'confidently placed in the TOL' or is there a more fundamental problem? It is probably a bit simplistic to conclude with the statement that 'this issue will be resolved by the incredible improvements in sequencing technology', when it has just been stated that the vast amounts of sequence are part of the problem. I suggest thinking this section through again with an eye to what is going on at deeper theoretical levels and in contrast (if possible) to what's going on in evolutionary microbiology. Avoiding the 'dewy mist' metaphor might be a good idea too.

*Conclusion*. As it stands, I think the concluding paragraph - which is just a repetition of 'there are problems but finding the natural unit of diversity is still the quest' - is a symptom of a general problem with the paper. The structure should lead to new insights for evolution and ecology (and beyond), not repetitions of what even newcomers to the field(s) know already. The point of comparing evolutionary microbiology and ecological microbiology should surely be to contrast more sharply their different approaches, show the major developments and problems, and to have some clear pointers of what might be needed to go beyond these respective problems. While contrasting them very strongly might be a bit artificial, it needs to be done for this paper otherwise it's déjà vu for the readers of *Biology Direct*.

**Author's response: **We thank the reviewer for her extensive comments and suggestions, which have greatly improved the manuscript. As a result, we have changed all three questions, altering them so they are more open ended. We have also corrected the use of "concepts" to that of "discoveries", as it is true that the topics discussed by the three questions are not concepts but rather major knowledge breakthroughs in microbiology. The importance of these discoveries is also explained after each question.

We acknowledge that the terms "Microbial Evolution" and "Microbial Ecology" linguistically refer to phenomena. However, the field decided to call itself "Microbial Ecology" (i.e. International Society for Microbial Ecology) and this is why we chose to use this term, and to be consistent, the term Microbial Evolution. We have changed the text to use the more accurate terms "evolutionary microbiology" and "ecological microbiology" wherever possible, while keeping the use of "Microbial Evolution" and "Microbial Ecology" when referring to those fields of science specifically.

The abstract and the conclusion have been completely re-written to emphasize the differences in the responses of evolutionary and ecological microbiology to major discoveries. This has also been done throughout the text.

We have changed the discussion about the validity of species definitions to one about their universality. We agree with the reviewer that the variation in causes of diversity has more relevance to questioning the universality of species definitions than its simple extent. However, we kept the third question about the extent of diversity (although in a format that more open ended and less presupposing of the conclusion), because we have as of yet no idea about how a variation in causes of diversity affects microbial ecology. Changing the question to be about causes rather than extent of diversity would have prevented a contrast to be drawn between the two branches of microbiology that are being discussed.
